# CTSV (cathepsin V) promotes bladder cancer progression by increasing NF-κB activity

**DOI:** 10.1080/21655979.2022.2061278

**Published:** 2022-04-20

**Authors:** Yue Xia, Minghuan Ge, Ling Xia, Guang Shan, Huijun Qian

**Affiliations:** a Department of Urology, Renmin Hospital of Wuhan University, Wuhan, Hubei, China; b Department of Radiation and Medical Oncology, Zhongnan Hospital of Wuhan University, Wuhan, Hubei, China

**Keywords:** CTSV, NF-κB, inflammation, bladder cancer

## Abstract

Chronic inflammation is positively associated with the development of urinary bladder cancer. However, its detailed regulatory mechanism remains elusive. The quantitative real-time polymerase chain reaction was used to measure mRNA levels of relative genes. The protein levels were monitored by western blotting. Cell proliferation and viability were evaluated by the cell counting Kit 8 (CCK8) and colony formation assays, respectively. The dual-luciferase reporter assay was performed to assay the transcriptional activity. *In vivo* experiments were implemented in nude mice as well. The TCGA database analysis suggested that the aberrant expression of cathepsin V (CTSV) was related to a poor outcome in bladder cancer patients. CTSV boosted the inflammation reaction, which facilitated the development of bladder cancer. The overexpression of CTSV increased the proliferation and viability of bladder cancer cells. On the contrary, the deletion of CTSV significantly inhibited the proliferation and viability of bladder cancer cells. The tumor repression resulting from CTSV deficiency *in vitro* was also verified *in vivo*. Moreover, multiple cancer-associated luciferase screening showed that the overexpression of CTSV triggered the inflammatory signaling pathway, which could be restored by introducing the NF-κB inhibitor. CTSV is upregulated and promotes proliferation through the NF-κB pathway in bladder cancer and may be a potential target in inflammation-associated bladder cancer.

## Graphical abstract

**Figure:**
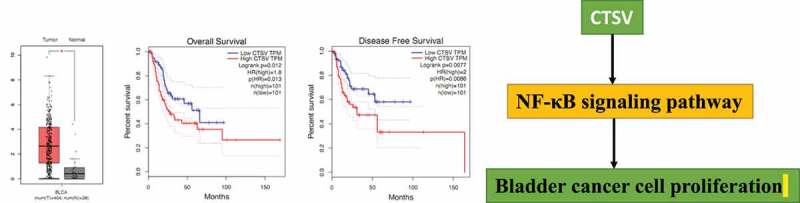

## Introduction

Bladder cancers (BCa) are estimated to be the tenth most common cancer found worldwide and the thirteenth most common cause of death from cancer, with almost 549,000 cases and 200,000 deaths reported in 2018[1]. Also, around 16% of individuals with bladder cancer were initially diagnosed with additional primary tumors in their lifetime. The incidence and mortality rates of BCa are about 9.6 and 3.2 per 100,000 men, respectively, which is about four times more than that found in women [2]. Cigarette smoking is the primary risk factor for bladder cancer, with 50% of cases attributed to smoking in both sexes [3–6]. It is also associated with various occupational events and environmental exposures. Additionally, metabolic changes have also been associated with the progression and proliferation of BCa [7]. Exposure to ionizing radiation or chronic inflammation is also an important risk factor [8–10].

As an essential host defense mechanism, inflammation is against an injury-caused infection in cells and organisms in response to stress. The immune system will eliminate the injury-causing stimuli when the regenerative and healing processes start [11–13]. However, excessive or persistent inflammation responses contribute to tumor development and progression by triggering a series of inflammatory cytokines and signal transduction. Chronic inflammation has been considered as one of the hallmarks of cancer [14–16]. The hypothesis that cancer originated from the sites of chronic inflammation was proposed in 1989[17], and since then, a large number of studies have been performed to investigate the association between the inflammatory microenvironment of malignant tissues and the development and progression of cancer. The accumulating body of evidence supports this hypothesis [18–22].

Cathepsin V (CTSV) is a type of lysosomal cysteine protease that belongs to the family of peptidase C1 [23,24]. It plays an important role in corneal physiology, and the activation of CTSV is effectively suppressed by various cysteine protease-specific inhibitors [25,26]. CTSV is aberrantly expressed in various tumors and plays a very important role in the breast ductal carcinoma processes [27,28], colorectal cancer [29–31], pancreatic cancer [32,33], liver cancer [34], and skin squamous cell carcinoma (SCC) [35–37]. CST6, a natural inhibitor of the lysosomal protease CTSV, inhibits bone metastasis of breast cancer by targeting CTSV [38,39], suggesting CTSV as a potential target in cancer treatment. Although the emerging evidence shows a pro-oncogenic role of CTSV in cancer development, its expression pattern and role in bladder cancer are still elusive.

Here, we hypothesized that CTSV plays a role during bladder carcinogenesis. We examined bladder cancer growth in vitro or in vivo when overexpressing or knocking out the CTSV. We also examined whether CTSV is a critical player during onco-inflammatory events. These findings suggest that CTSV might be a potential target for the treatment of BCa patients by changing the inflammatory pathway.

## Materials and methods

### Cell culture and cell lines

The UMUC3 and T24 cell lines and HEK293T cell lines were obtained from the Cell Bank of Type Culture Collection of the Chinese Academy of Sciences. The cells were cultured in Dulbecco’s modified Eagle’s medium (DMEM) (Gibco) supplemented with 10% (v/v) fetal bovine serum (FBS; HyClone, Logan, UT) and 100 U penicillin-streptomycin (Gibco, Gaithersburg, MD, USA) and were incubated in a cell culture incubator at 37°C with 5% CO_2_. Additionally, the cell lines EJ and 5637 were cultured in RPMI-1640 medium containing 10% (v/v) fetal bovine serum and 100 U penicillin-streptomycin (Gibco, Gaithersburg, MD, USA) and incubated in a cell culture incubator at 37°C with 5% CO_2_. CTSV-specific knockout was generated in the T24 cells by CRISP/Cas9 mediated gene deletion [40]. The sgRNA sequence is listed as follows: 5’-GAATACAGCCAAGGGAAACA-3'. For CTSV overexpression, the cDNA of CTSV gene were amplified and cloned into pLV-Flag (Inovengen Tech. Co, China) [41]. The recombinant constructs and the empty pLV-Flag vectors were produced packaged in 293 T cells. The resulted viral particles were transduced into UMUC3 cells after concentration and purification. Next, the UMUC3 cells were subjected to 14-day puromycin maintenance. The survival clones were obtained and amplified. Western blot and Sanger sequence were performed to verify CTSV overexpression.

### Quantitative PCR analysis

The cells were cultured until they achieved 90% confluence and then collected after the TNFα (20 ng/ml,abclone,Cat#:RP00001) treatment at the indicated time. The cDNA was generated using reverse-transcriptase PCR, which was further used for the quantitative PCR reactions. The used primer sequences are as follows: IκBα-forward (5'-GATCCGCCAGGTGAAGGG-3') and reverse (5'-GCAATTTCTGGCTGGTTGG-3'); TNFα-forward (5'-GCCGCATCGCCGTCTCCTAC-3') and reverse (5'-CCTCAGCCCCCTCTGGGGTC-3'); GAPDH-forward (5'-GAGTCAACGGATTTGGTCGT-3') and reverse (5'-GACAAGCTTCCCGTTCTCAG-3'). The data was analyzed with the Delta-Delta CT method [42].

### Western blot analysis

Cells were cultured until they were approximately 90% confluent, then lysed with a sodium dodecyl sulfate (SDS) lysis buffer (50 mM Tris-HCl-pH 6.8, 10% glycerol, 2% SDS) supplemented with a protease inhibitor cocktail (Roche, 04693132001). The whole-cell lysates were separated using SDS/PAGE gels and further visualized by enhanced chemiluminescence. Different antibodies used to detect the target proteins are as follows: The Rabbit Polyclonal Antibody CTSV (A7662, dilution 1:1000), Rabbit Polyclonal Antibody IκBα (A1187, dilution 1:1000), Rabbit Monoclonal Antibody GAPDH (A19056, dilution 1:5000), and Mouse Monoclonal Antibody Flag (A7662, dilution 1:5000). These antibodies were purchased from the ABclonal Company (Wuhan, Hubei, China).

### Cell growth and colony formation assays

Cell Counting Kit-8 (CCK-8) was used to evaluate the cell viability following the standard procedure of the kit. All the cells (2 × 10^3^) were plated into a 96-well plate. After the cell growth, 100 µL of fresh complete DMEM medium with 10% CCK-8 reagent was added to the plate, which was then incubated in a CO_2_ incubator at 37°C for 1.5 hours. Next, the ELx800 microplate reader (BioTek, USA) was used to measure the absorbance of each well at 450 nm. The absorbance value was considered proportional to the cell viability *[43]*. For the colony formation assay *[44]*, around 800 UMUC3 or T24 cells were seeded in a 6-well plate, and after about 12 days, the cell colonies were stained with 0.025% crystal violet and photographed to count the number.

### Mouse xenograft assays

Pathogen-free 5-week-old female C57BL/6 mice were purchased from the Animal Resource Center at the Wuhan Institute of Virology, Chinese Academy of Sciences, China. A total of 14 mice were caged individually and provided free access to food and water under controlled lighting conditions. CTSV-deficient T24 cells and the control cells were implanted into mice. The mice received the measurement every four days. On the 31^st^ day, the mice were sacrificed by CO_2_ inhalation, and the weight was recorded [45]. Ethical approval was obtained from the Committee on Ethics in the Care and Use of Laboratory Animals of Wuhan, China. All protocols were conducted in accordance with the Guidelines of the China Animal Welfare Legislation.

### Luciferase assay

HEK293T cells (1 × 10^5^) were inoculated into a 24-well plate and incubated till the cells adhered to the plate, after which PEI was used for transfection. The corresponding reporter plasmids, NF-κB and pRL-TK, and the target gene plasmid were included in each transfection system. Once stable expression was achieved, the fluorescence intensity of the reporter genes was measured using the dual-luciferase detection kit. The intensity of NF-κB fluorescence was proportional to the intensity of the pRL-TK reporter gene expression *[46]*.

### Statistical analysis

All results were analyzed using GraphPad Prism or SPSS software. The values were presented as the mean ±SD. Differences between the two groups were analyzed using an unpaired Student’s t-test. For multiple group comparisons, one-way ANOVA was employed followed by Bonferroni post hoc analysis. P < 0.05 was considered statistically significant.

## Results

Here, we demonstrated that CTSV facilitated cell proliferation and viability by overexpressing or knocking out the CTSV gene in different bladder cancer cell lines. The *in vitro r*esults were further validated *in vivo*. Additionally, CTSV is also involved in triggering the inflammatory signaling pathway, and its high expression in bladder cancer patients indicates a poor prognosis.

## High expression of CTSV in bladder cancer

**Figure 1.**
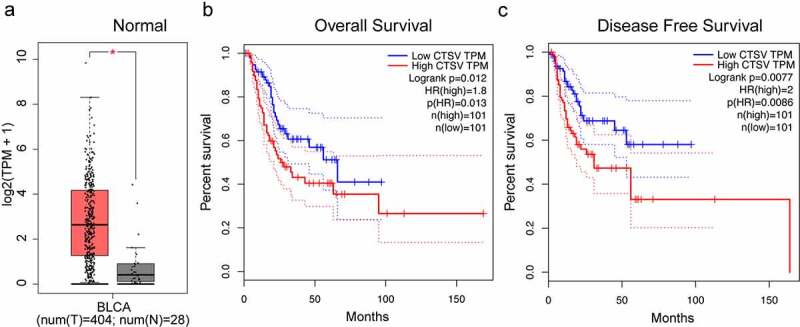
Aberrant expression of CTSV in bladder cancer. A. The relative mRNA expression of CTSV in the bladder cancer tissues (n = 404) and normal tissues (n = 26). B. A Kaplan-Meier survival curve showing a significant association between the high levels of CTSV and poor overall survival of bladder cancer patients (p = 0.013). Low CTSV (n = 101) and high CTSV (n = 101). C. A Kaplan-Meier survival curve showing a significant association between the high levels of CTSV and poor survival in disease-free bladder cancer patients (p = 0.0086). Low CTSV (n = 101) and high CTSV (n = 101). Statistical significance was determined by Student’s t-*test*. *, p < 0.01.

We performed an mRNA expression level analysis based on the TGCA and GTEx databases from the GEPIA (Gene Expression Profiling Interactive Analysis) website to evaluate the CTSV expression patterns in bladder cancer. The results showed that CTSV-mRNA expression was upregulated in the bladder cancer tissues compared to that of the normal tissues (Figure 1a). To test whether the increased mRNA level of CTSV was associated with poor prognosis in bladder cancer, we carried out Kaplan–Meier analysis from GEPIA online tool. The results showed that the bladder cancer patients with high CTSV levels had remarkably poorer overall and disease-free survival rates than the patients with lower levels of CTSV (Figures 1b and 1c), suggesting a potential role of CTSV in the development and progression of bladder cancer. Overall, our findings indicated that CTSV was expressed abnormally in bladder cancer tissues and that high CTSV expression was associated with a poor prognosis in bladder cancer patients.

## Overexpression of CTSV facilitates the cell viability of bladder cancer

**Figure 2.**
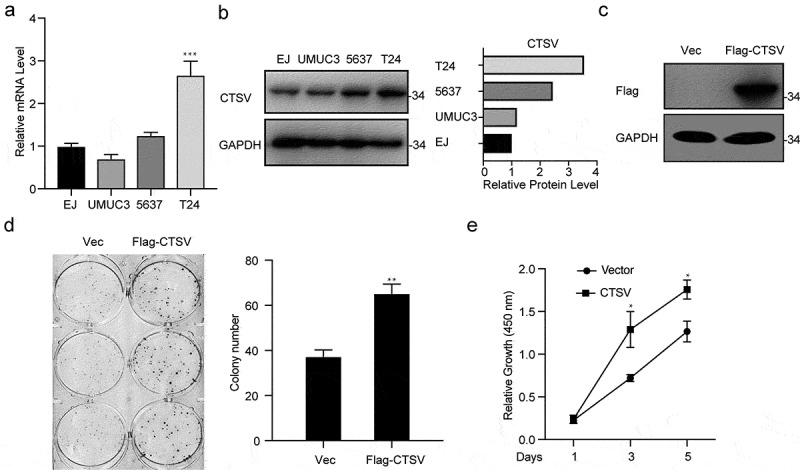
Overexpression of CTSV facilitates bladder cancer cell viability. A and B. The qPCR and western blotting of the relative CTSV mRNA expression and protein levels, respectively in different bladder cancer cell lines, including EJ, UMUC3, 5637, and T24. UMUC3 was used to construct the CTSV overexpression cell line. C. The CTSV expression in UMUC3 cell lines tested by immunoblotting using an anti-Flag antibody. D. Colony formation assay performed both in wild-type cells and CTSV overexpression stable cell line with quantification of the colony numbers. E. Cell growth curve assay performed in wild-type cells and CTSV overexpression stable cell line. The relative cell number was measured at 450 nm using the CCK8 method on the indicated days. Error bars and mean ± SD obtained from three replicates. Statistical significance was determined by Student’s t-*test* or one-way ANOVA, *, p < 0.05, **, p < 0.01, ***, p < 0.001.

To determine the function of CTSV in the progression of bladder cancer, we first quantified the CTSV expression in a pan of bladder cells. As shown in Figure 2a, UMUC3 cells exhibited the lowest mRNA expression of CTSV. The low protein levels of CTSV in UMUC3 were determined by the western blotting test (Figure 2b). The stable overexpression of CTSV in UMUC3 cells was established for the functional assays (Figure 2c). To assess the effects of CTSV on cell viability and growth, colony formation and growth curve assays were employed. The results showed that the overexpressed CTSV remarkably increased the colony number and size, suggesting that CTSV facilitated bladder cancer cell viability (Figure 2d). Meanwhile, the cell growth curve assay also showed that CTSV increased the cell growth ability, suggesting CTSV’s role in enhancing the cell proliferation in bladder cancer (Figure 2e). Hence, our results indicated that CTSV was crucial for enhancing cell viability and proliferation in bladder cancer.

## CTSV knockout represses the tumorigenesis *in vitro* and *in vivo*

**Figure 3.**
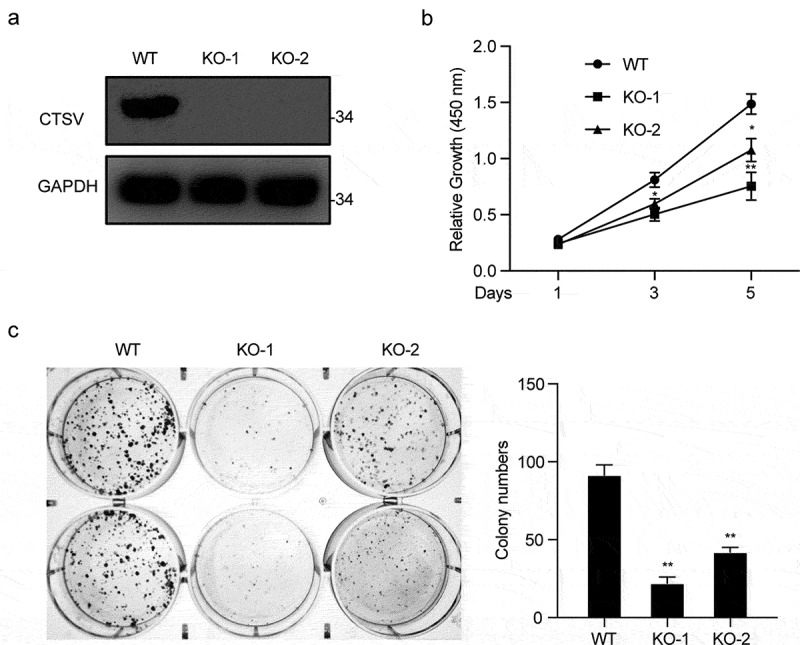
CTSV deficiency curbed *in vivo* xenograft tumor growth of T24 cells. CTSV-deficient T24 cells or the negative control were implanted into the right flank of each nude mouse to generate tumors. A. The photography of xenograft tumors after 31 days subcutaneous implantation of the T24 cells. B. CTSV expression in xenograft tumor tissues was detected by western blot. C.Weight of xenografted tumors at 31 days post-transplantation. D. Subcutaneous tumor growth curves. ****, p < 0.0001.

**Figure 4.**
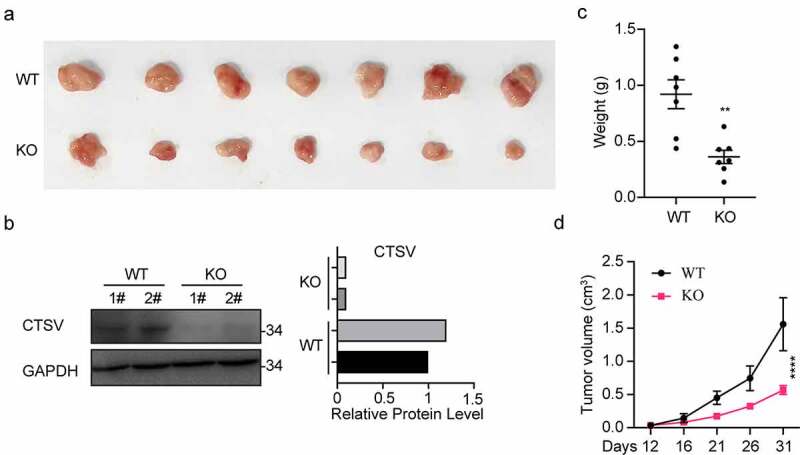
CTSV knockout represses tumorigenesis in vitro. A. CTSV gene inactivation in T24 bladder cancer cell line via CRISPR/Cas9 mediated somatic cell knockout method. The expression of CTSV protein in wild-type and knockout cells was determined by immunoblotting. B. Colony formation assay performed in wild-type cells and targeted knockout CTSV cells and the quantification of their colony numbers. C. Cell growth curve assay performed in wild-type cells and targeted knockout CTSV cells. The relative cell number was measured at 450 nm by the CCK8 method on the indicated days. Error bars and mean ± SD obtained from three replicates. Statistical significance was determined by Student’s t-*test* or one-way ANOVA, **, p < 0.05, **, p < 0.01.

To explore tumorigenesis in CTSV-deficient bladder cancer cells, the CTSV gene expression was reduced in the T24 bladder cancer cell line via the CRISPR/Cas9 mediated gene knockout. The immunoblotting analysis detected the expression of CTSV protein both in wild-type and targeted knockout cells, which showed a successful depletion of CTSV in the T24 bladder cancer cell line (Figure 3a). To assess the influence of CTSV on cell viability and cell growth, the colony formation and growth curve assays were employed. The results showed that CTSV-depleted cells could significantly decrease the colony number and size, suggesting that its deficiency could cause attenuation in the bladder cancer cell viability (Figure 3b). Besides, the cell growth curve assay showed that CTSV-knockout decreased the cell growth ability, thus, repressing bladder cancer cell proliferation (Figure 3c). More importantly, the reduction in tumorigenic potential caused by CTSV deficiency was witnessed *in vivo*, evidenced by smaller tumor size and weight in mice bearing CTSV-deficient T24 cells compared with the control groups (Figure 4a-d). In summary, our data showed that CTSV is an oncogene *in vitro* and *in vivo*.

## CTSV promotes NF-κB inflammatory signaling pathway

**Figure 5.**
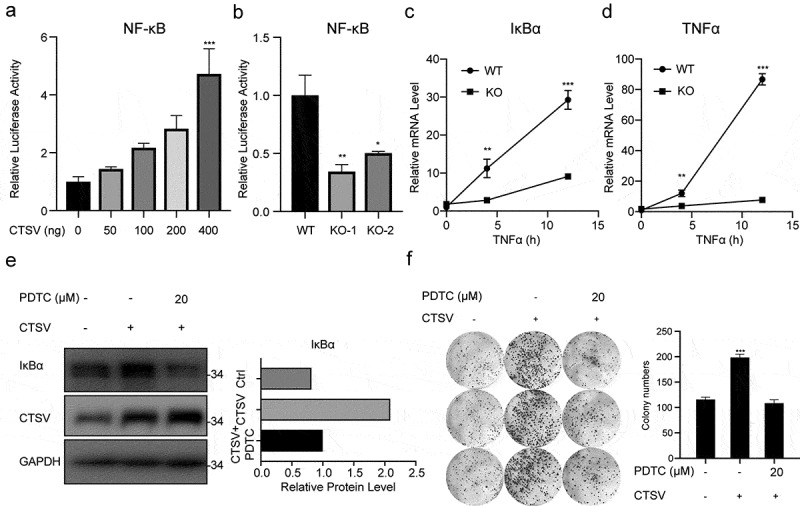
CTSV resulting in the NF-κB signaling pathway. A. 293 T cells were transfected using 200 ng of NF-κB luciferase reporter plasmid along with the CTSV expression plasmid in different doses or equivalent empty vector. Whole-cell extracts were used for the luciferase assay. B. NF-κB luciferase reporte*r plasmid* was introduced into CTSV-deficient cells. Whole-cell extracts were used for the luciferase assay. C. qPCR analysis of the relative IκBα in wild-type and targeted knocked-out CTSV-T24 cells treated with TNFα (20 ng/ml, abclone, cat#: RP00001) at different times. D. RT-qPCR analysis of TNFα mRNA expression in wild-type and targeted knocked-out CTSV-T24 cells treated with TNFα (20 ng/ml, abclone, cat#: RP00001) at different times.E. Immunoblotting of the indicated protein in CTSV-overexpressed UMUC3 cells with and without PDTC. F. Colony formation assays in both wild-type cells and CTSV-overexpressed UMUC3 cells with and without PDTC with the quantification of colony numbers. Error bars and mean ± SD obtained from three replicates. Statistical significance was determined by Student’s t-*test* or one-way ANOVA, *, p < 0.05, **, p < 0.01, ***, p < 0.001.

We performed the dual-luciferase reporter analysis in the 293 T cell line to test whether CTSV affected the inflammatory signaling pathway. The analysis showed that CTSV increased NF-κB transcriptional activation in a dose-dependent way, which indicated CTSV’s positive association with NF-κB transcriptional activity (Figure 5a). The declined NF-κB transcriptional activity was also detected in CTSV-deficient cells (Figure 5b). To further confirm the effect of CTSV on NF-κB activation in bladder cancer cells, TNFα was used to activate the NF-κB pathway in CTSV-deficient cells. The qPCR data showed that the mRNA level of NF-κB downstream genes, i.e., IκBα and TNFα showed dramatic elevation in CTSV-deficient T24 cells when compared to that of the wild-type cells (Figures 5c and 5d). Consistent with the mRNA level, western blotting also showed an increased protein level of IκBα upon CTSV overexpression, which was rescued by the NF-κB pathway inhibitor pyrrolidine dithiocarbamate ammonium (PDTC), suggesting that CTSV specifically triggered the NF-κB inflammatory signaling pathway (Figure 5e). Furthermore, to confirm the involvement of the NFκ-B pathway in CTSV-induced cell viability increase in bladder cancer, we used PDTC to pretreat the bladder cancer cells before performing the colony formation assay. The result showed that CTSV induced increase in the colony number was reduced by PDTC, which indicated that the oncogenic role of CTSV was mediated through a specific increase in the activity of the NF-κB pathway (figure 5f). Overall, CTSV promoted bladder cancer cell viability by facilitating the activity of the NF-κB inflammatory signaling pathway.

## Discussion

Bladder cancer is one of the most common urinary tract tumors found in the world [47]. Although often diagnosed at an early stage, the relapse of bladder cancer is quite high [48]. Bladder cancer development mechanism and the relevant markers are poorly understood, which limits its effective prevention and treatment [49]. Therefore, our study aimed to find important genes in bladder cancer development and progression.

As per our knowledge, CTSV is a type of lysosomal cysteine protease belonging to the peptidase C1 family and is highly upregulated in colorectal and breast carcinomas compared to normal tissues. Although the emerging evidence shows a pro-oncogenic role of CTSV in cancer development, the biological functions of CTSV in bladder cancer have not been characterized yet. Here, we found that CTSV is dramatically increased in bladder cancer tissues when compared to normal tissues. Kaplan-Meier data showed that a higher CTSV expression could decrease the overall survival and disease-free survival of BCa patients, indicating an important role of CTSV in bladder cancer progression, which could be used as a potential prognostic biomarker and therapeutic target in bladder cancer. Our results were also consistent with the prognostic significance of CTSV in HCC and breast cancer [27,50]. We firstly assessed the expression of CTSV in bladder cancer cells. We noted that CTSV mRNA was downregulated in UMUC3 cells, while its protein expression showed no difference with that of control cells. Its expression showed a different trend in bladder cancer cells. This differential expression might indicated the different regulatory mechanism behind CTSV expression. To study the biological role of CTSV in the development and progression of bladder cancer, we constructed a stably expressing CTSV-UMUC3 cell line. The colony formation and growth curve assays showed that CTSV overexpression could increase the bladder cancer cell viability and proliferation.

On the other hand, CRISPR/Cas9 mediated CTSV silencing in the T24 bladder cancer cell line attenuated the cell viability and proliferation in the bladder cancer that had high expression of CTSV. As a result, it is proposed that CTSV depletion could inhibit the tumorigenesis ability of bladder cancer cells. In short, our data showed that CTSV as an oncogene contributed to the proliferation and viability in bladder cancer cell lines. To now, no adequate research has been carried out to investigate the function of CTSV in cancer. Thus, through the overexpression and deletion of CTSV, we are the first to clarify the role of CTSV in bladder cancer development and progression, suggesting CTSV to be a potential target in bladder cancer.

Inflammation is shown to be closely related to cancer development and progression. Proinflammatory factors such as IL-1, IL-6, IL-8, and TNFα generally enhance the occurrence and development of cancer by activating growth, metastasis, and invasion [51]. Indeed, Anti-CTSV treatment has anti-inflammation effect in the thoracic aorta of hyperhomocysteinemia mice [52]. CTSV expression is associated with IL-17A stimulation, which is pro-inflammatory [53]. The NF-κB pathway links inflammation to cancers [54,55]. Therefore, we employed the dual-luciferase assay to determine whether CTSV is associated with oncogenic NF-κB pathway. The results just showed that CTSV was associated with the NF-κB inflammation pathway. As expected, CTSV overexpression resulted in increased activity of the NF-κB pathway, and we further confirmed this association using the qPCR analysis in CTSV-silenced bladder cancer cells. Moreover, PDTC, a specific target in the NF-κB signaling, inhibited CTSV induced NF-κB activation and bladder cancer cell growth. These results suggested that the oncogenic role of CTSV is through the activation of the NF-κB signaling in bladder cancer. We concentrated on the in vitro phenotype and did not look at the in vivo function. Furthermore, the mechanism by which CTSV regulated the NFκ-B signaling pathway has not been elucidated. As a result, more rigorously designed experiments are required to demonstrate CTSV’s role in BCa.

## Conclusion

In summary, our data demonstrated that CTSV is an important oncogene that participated in the development and progression of bladder cancer. CTSV is also crucial for cancer cell viability and proliferation since it triggers the inflammatory pathway. Our study suggested a pro-oncogenic role of CTSV in bladder cancer, which could be used as a biomarker and potential target in the study of bladder cancer.
